# Effectiveness of free vastus lateralis musculocutaneous flap transplantation plus ultrasound-mediated transdermal Qianjin Weijing decoction for chronic empyema: a prospective, preference-based observational study

**DOI:** 10.3389/fmed.2026.1780744

**Published:** 2026-03-12

**Authors:** Fei Chen, Chao Wang, Zhihan Wang

**Affiliations:** 1 Department of Cardiothoracic Surgery, Tongde Hospital of Zhejiang Province, Hangzhou, Zhejiang, China; 2 Department of Cardiology, Taizhou Central Hospital, Taizhou, Zhejiang, China

**Keywords:** anterolateral thigh flap, chronic empyema, myocutaneous flap, prospective observational study, ultrasound-mediated drug delivery

## Abstract

**Objective:**

To evaluate the effectiveness of free vastus lateralis musculocutaneous flap (VLMCF) transplantation combined with ultrasound-mediated delivery of Qianjin Weijing decoction for chronic empyema.

**Methods:**

In this single-center prospective observational study (July 2023–June 2025), 114 patients elected either VLMCF surgery alone (control, *n* = 57) or VLMCF plus ultrasound-assisted transdermal decoction at bilateral Feishu points for 2 weeks (combined, *n* = 57). The primary outcome was residual cavity volume measured via 3D-CT. Secondary outcomes included nutritional, inflammatory, and quality-of-life (SF-36) markers. Analyses used ANCOVA to adjust for baseline imbalances and multiple imputation for missing data.

**Results:**

At 2 weeks, the combined-therapy group showed a significantly greater reduction in cavity volume compared to the control (20.52 \ ± 4.45 vs. 24.40 \ ± 6.35 mL; adjusted mean difference: −3.72 mL; 95% CI: −5.85 to −1.59; *p* = 0.005). By 3 months, cavity volumes were comparable. Regarding secondary outcomes, the combined group had significantly lower procalcitonin levels at 3 months (adjusted difference: −0.10; *p* = 0.0005). However, no robust differences were found in albumin, hemoglobin, CRP, WBC, or functional recovery (ADL/SF-36) at either time point after FDR correction.

**Conclusion:**

Adding ultrasound-mediated Qianjin Weijing decoction to VLMCF transplantation may accelerate early cavity resolution and improve procalcitonin profiles in chronic empyema patients. While mid-term functional and nutritional outcomes were similar to surgery alone, the combined regimen is a safe potential adjunct. Randomized controlled trials are needed to confirm long-term benefits and causality.

## Introduction

Chronic empyema is a debilitating sequela of pleural infection characterized by persistent pleural space, fibrous peel formation, and failure of lung re-expansion despite prolonged drainage and antibiotic therapy. Patients often present with recurrent fever, chest pain, dyspnea, and marked impairment in nutritional status and functional capacity (1). Although advances in thoracic surgery and perioperative care have improved the prognosis of empyema, the management of chronic empyema with organized pleural cavity remains challenging, particularly in individuals with large residual spaces, thick fibrotic pleura, and compromised pulmonary reserve (2).

For selected patients with chronic empyema, radical debridement combined with thoracoplasty or muscle flap transposition is considered a definitive surgical option. Free or pedicled muscle or musculocutaneous flaps, such as the latissimus dorsi or vastus lateralis flap, can effectively obliterate the residual cavity, provide well-vascularized tissue to control infection, and reinforce the chest wall (3). However, even after technically successful flap surgery, some patients experience delayed cavity closure, prolonged drainage, persistent low-grade infection, and slow recovery of systemic inflammation and lung function. Optimizing local infection control and promoting cavity obliteration in the early postoperative period therefore remain key goals to improve long-term outcomes (4).

Traditional Chinese medicine (TCM) has a long history of treating purulent lung and pleural diseases, including conditions corresponding to “Fei Yong” (pulmonary abscess) described in classical texts. Qianjin Weijing Decoction is a classical formula originally prescribed for pulmonary abscess and related suppurative disorders and has more recently been applied to a range of respiratory diseases, such as pneumonia, chronic obstructive pulmonary disease and air-pollution–related lung injury, often as an adjunct to conventional therapy (5). In modern clinical practice, Weijing-based formulas have been administered orally or used in external preparations to support the resolution of pulmonary infections and pleural effusions (5). However, we acknowledge that direct, high-quality clinical evidence specifically supporting Qianjin Weijing Decoction for empyema—particularly in the postoperative setting—remains limited. Ultrasound-facilitated transdermal drug delivery (sonophoresis or phonophoresis) is a non-invasive technique that uses ultrasound energy to increase skin permeability and enhance the percutaneous absorption of topical agents, thereby potentially increasing local drug concentrations while limiting systemic exposure (6). Experimental and early clinical studies have shown that ultrasound-enhanced transdermal delivery can augment local anti-inflammatory effects for several drugs, although high-quality evidence remains limited for many indications (7). Combining a Qianjin Weijing–based decoction with aatherefore provide an additional means of modulating the local inflammatory milieu and promoting residual cavity resolution. However, rigorous clinical data on such integrative approaches in chronic empyema are scarce, and well-designed randomized controlled trials are still lacking.

Free vastus lateralis musculocutaneous flap (VLMCF) transplantation has been increasingly used to reconstruct complex chest wall defects and obliterate chronic empyema cavities. We hypothesized that adding ultrasound-assisted transdermal administration of Qianjin Weijing Decoction in the early postoperative period could further accelerate cavity reduction, enhance infection control and nutritional recovery, and improve functional and quality-of-life outcomes without compromising safety. Therefore, we conducted a prospective, single-center observational study in which treatment allocation was based on patient preference after full clinical consultation. The study compared patients receiving free VLMCF transplantation alone with those who elected to receive additional ultrasound-mediated transdermal delivery of Qianjin Weijing Decoction during postoperative care. The primary objective was to evaluate differences in the rate of empyema cavity volume reduction at 2 weeks, 3 months after surgery between the two treatment strategies. Secondary objectives were to assess radiological outcomes, inflammatory and nutritional recovery, pulmonary function, health-related quality of life, and safety profiles over the follow-up period.

Feishu (BL13), the back-shu point of the Lung, is one of the most commonly selected acupoints in acupuncture/acupoint-based interventions for respiratory disorders (e.g., asthma and chronic obstructive pulmonary disease). Systematic reviews and meta-analyses of acupuncture for chronic respiratory diseases have reported improvements in symptom burden and quality-of-life outcomes, although methodological quality and heterogeneity vary across trials; notably, BL13 is frequently included in respiratory acupoint prescriptions. Mechanistic studies further suggest that stimulation at BL13 may be associated with modulation of immune and cytokine-related pathways relevant to respiratory inflammation (8, 9). Importantly, high-quality clinical evidence specifically supporting acupoint-based interventions in chronic empyema—particularly in the postoperative setting—remains scarce. Therefore, in the present study, BL13 was primarily used as a standardized anatomical application site for ultrasound-facilitated transdermal delivery to enhance procedural consistency and reproducibility, rather than as a stand-alone needle acupuncture intervention.

## Methods

### Study design and setting

This was a prospective, single-center observational study conducted in the Department of Thoracic Surgery at Tongde Hospital of Zhejiang Province, China. The study period was from July 1, 2023 to June 30, 2025 and included patient enrollment, surgical treatment, postoperative interventions, follow-up assessments, and data analysis. The study compared outcomes between patients who underwent free VLMCF transplantation alone and those who voluntarily chose to receive additional ultrasound-assisted transdermal administration of Qianjin Weijing Decoction during postoperative care. Treatment allocation was based on patient preference after standardized clinical consultation regarding both therapeutic options, potential benefits, and risks.

The study protocol was approved by the institutional ethics committee of Tongde Hospital of Zhejiang Province (approval No.2023-078(K)). All participants provided written informed consent prior to enrollment. The study followed a prospective observational design where treatment was allocated based on patient preference after full clinical consultation. The study was conducted in accordance with the Declaration of Helsinki and relevant national regulations.

### Diagnostic criteria for chronic empyema

Chronic empyema was diagnosed based on a combination of clinical, radiological, and laboratory criteria, consistent with contemporary thoracic surgery and respiratory medicine guidelines:1. Clinical manifestations: long-standing symptoms such as fever, chest pain, chronic cough, purulent sputum, and/or fatigue. 2. Imaging findings: chest computed tomography (CT) showing pleural thickening, fibrous peel formation, and a loculated empyema cavity. 3. Pleural fluid characteristics: purulent pleural effusion obtained through chest tube drainage or thoracentesis, with supportive microbiological evidence when available. 4. Disease duration: ≥3 months from initial onset of pleural infection or empyema to enrollment.

### Inclusion criteria

Adults aged 18–70 years; clinically and radiologically confirmed chronic empyema with disease duration ≥3 months; presence of organized cavity with fibrous peel and insufficient response to conventional treatments (antibiotics, drainage, and supportive care); deemed suitable for radical surgical management using a free VLMCF by a multidisciplinary team; American Society of Anesthesiologists (ASA) physical status class I–III; able to comply with postoperative follow-up for ≥3 months; signed informed consent.

### Exclusion criteria

Severe cardiac, hepatic, or renal dysfunction (e.g., NYHA class III–IV heart failure, Child–Pugh class C cirrhosis, end-stage renal disease); clinically significant coagulation disorders or immunodeficiency; pregnancy or lactation; known allergy to components of Qianjin Weijing Decoction or conductive materials; severe psychiatric disorders affecting cooperation; advanced malignant disease with life expectancy <1 year; participation in another interventional clinical study within 1 month before enrollment.

### Discontinuation criteria

Patients were excluded from the per-protocol analysis if: the planned surgical procedure was significantly modified intraoperatively; severe complications required emergency deviation from the protocol; consent was withdrawn or study treatment was discontinued by request; postoperative pathological or clinical assessment contradicted the diagnosis; loss to follow-up occurred (two consecutive missed visits without contact).

Such cases remained included in the full analysis set, with appropriate methods applied to address missing data.

### Blinding

Due to the inherent differences between treatment options and patient-driven allocation, blinding of participants and surgeons was not feasible. However, radiologists performing cavity volume measurements investigators conducting questionnaire evaluations, and statistical analysts remained blinded to treatment grouping throughout data acquisition.

### Interventions

#### Surgical procedure (both groups)

All enrolled patients underwent standardized free VLMCF transplantation performed by the same experienced surgical team following a unified institutional operative protocol. Briefly, thorough decortication and debridement of the empyema cavity were performed through appropriate thoracic access to remove fibrous peel, necrotic tissues, and infectious material, while facilitating maximal lung re-expansion. The flap was designed and harvested from the lateral thigh based on preoperative planning to ensure adequate muscle bulk and a suitable skin paddle to obliterate the thoracic cavity and reconstruct the chest wall defect. After transplantation, the flap was inset to fill the cavity, microvascular anastomosis was completed to recipient vessels, and thoracic as well as donor-site drainage tubes were placed with negative-pressure drainage according to institutional standards. Both donor and recipient sites were closed in layers.

Postoperatively, all patients received standardized care including: systemic antibiotic therapy guided by microbiological results (typically for 2–4 weeks), analgesia, nutritional support, respiratory physiotherapy, and routine monitoring of flap viability, wound status, drainage, and dressings according to a predefined protocol.

#### Combined-therapy group (Group 1)

Patients who elected to receive integrative therapy underwent the same surgical procedure and postoperative routine care as the surgery-alone group, with the addition of ultrasound-assisted transdermal administration of Qianjin Weijing Decoction.

#### Qianjin Weijing decoction formula and preparation

Qianjin Weijing decoction was prepared using a fixed formula: Phragmitis rhizoma 60 g; Coicis semen 30 g; Platycodonis radix 30 g; Benincasae semen 24 g; Houttuyniae herba 20 g; Taraxaci herba 20 g; Prunellae spica 20 g; Lonicerae japonicae flos 15 g; Forsythiae fructus 10 g; Fritillariae bulbus 10 g; Glycyrrhizae radix et rhizoma 10 g; Platycladi cacumen 10 g; Persicae semen 9 g, and was concentrated to a final volume of 100 mL per batch.

All crude herbs were dispensed by the hospital pharmacy, and supplier information and batch numbers were recorded for traceability. The decoction was prepared following a standardized workflow (soaking → decoction → filtration → concentration) to reach the target final volume.

### Quality control

Chromatographic fingerprinting/marker quantification was not performed in this clinical study; this has been stated explicitly as a limitation. However, all herbs were supplied under routine pharmacy quality standards and batch traceability was ensured.

Ultrasound-assisted transdermal administration: Ultrasound-assisted transdermal drug delivery was administered using a pulsed ultrasound–electroconductive therapy device (SLC-005), applied bilaterally to standardized upper-thoracic paraspinal skin sites at the T3 vertebral level (approximately 2–3 cm lateral to the posterior midline). Treatment sites were identified using anatomical landmarks by trained study personnel (7, 10). Route of administration: topical/transdermal (percutaneous) delivery via sonophoresis/phonophoresis over bilateral BL13; no oral or intravenous administration of Qianjin Weijing Decoction was used in this study.

Beginning on postoperative day 2, patients received one session daily for 14 consecutive days (one 2-week course). During each session, the patient was positioned to expose the upper back; the skin over BL13 was cleansed and inspected; an appropriate amount of Qianjin Weijing decoction was applied to the treatment area; and the transducer was moved slowly to maintain uniform contact. A coupling medium (conductive gel) was used when needed to ensure adequate acoustic/electrical contact.

Device settings followed the manufacturer’s standard pulsed mode for transdermal delivery. The current index was set at 5–20 mA and adjusted to patient tolerance (mild warmth/tingling without pain). Each session lasted 30 min. An adherence-restricted (per-protocol) cohort was defined *a priori* (completion of ≥10 ultrasound-assisted sessions in the combined-therapy group and no receipt of adjunct therapy in the surgery-alone group, with completed follow-up assessments) and was used for sensitivity analyses.

Any discomfort potentially related to treatment (local burning, intense pain, palpitations, chest tightness, erythema, blistering) prompted immediate suspension or termination of the session and was documented as a potential adverse reaction. Sessions were not performed on areas with dermatitis, wounds, or broken skin.

To avoid confounding effects, other external TCM therapies with anti-inflammatory or blood-activating actions (herbal plasters or external devices) were not permitted during the study period. If oral TCM medication was used for concomitant conditions, its formula, dosage, and duration were recorded.

### Surgery-alone group (Group 2)

Patients who selected surgery alone received only the aforementioned operative procedure and routine postoperative care. No Qianjin Weijing Decoction or ultrasound-assisted transdermal therapy was administered.

### Outcomes

#### Primary outcome

The primary outcome was the absolute residual cavity volume at 2 weeks and 3 months after surgery, measured by three-dimensional CT reconstruction. CT scans were acquired using the same model of spiral CT scanner (slice thickness ≤1.5 mm). Residual cavity volume was quantified using dedicated 3D reconstruction software (Mimics). Two experienced thoracic radiologists, blinded to combined-therapy group, independently delineated and measured the cavity volume. The mean value was used for analysis. Inter-observer consistency was assessed using the intraclass correlation coefficient (ICC). When discrepancies exceeded 10%, a third senior radiologist adjudicated the final measurement.

#### Secondary outcomes

Laboratory parameters: Inflammatory markers: C-reactive protein (CRP) (11), procalcitonin (PCT) (7, 12), white blood cell count (WBC). Nutritional markers: serum albumin (ALB).

All were assessed at baseline and at postoperative weeks 2, and 3 months. These biomarkers were interpreted as supportive evidence and were not used as the primary basis for efficacy conclusions.

Quality of life: Activities of daily living (ADL) assessed using the Barthel Index (BI), [Chinese validated version], administered by trained study staff at baseline, 2 weeks, and 3 months. The BI comprises 10 items and yields a total score ranging from 0 to 100, with higher scores indicating greater independence. For participants with partial item nonresponse, BI total scores were calculated only when ≥ [8/10] items were completed; otherwise the BI score was treated as missing and handled using multiple imputation as described below.

#### Health-related quality of life

Health-related quality of life was measured using the 36-Item Short Form Health Survey (SF-36), [version: SF-36 v1], [language: validated Chinese versio] at baseline, 2 weeks, and 3 months. SF-36 domain scores were transformed to a 0–100 scale according to standard scoring procedures, with higher scores indicating better health status. In addition to domain scores, we calculated the Physical Component Summary (PCS) and Mental Component Summary (MCS) scores using the norm-based scoring algorithm for the [Chinese population norms]. For missing SF-36 items, scoring followed the standard SF-36 rule-based approach (domain scores were computed when at least 50% of items within a domain were completed); otherwise the corresponding domain and summary scores were treated as missing and addressed using multiple imputation.

### Safety and complications

All adverse events (AEs) were recorded throughout the study period, with specific attention to events potentially related to ultrasound-assisted therapy or the herbal solution (e.g., local erythema, burning pain, blistering, palpitations, and chest tightness). Serious adverse events (SAEs) were defined as events resulting in death, life-threatening conditions, hospitalization or prolonged hospitalization, persistent or significant disability/incapacity, or congenital anomaly/birth defect. Surgical complications (e.g., partial flap necrosis, wound infection, bleeding/hematoma, chest-wall deformity/asymmetry, and respiratory failure) were recorded. Safety outcomes were evaluated over two prespecified time windows: (i) from surgery to postoperative week 2, and (ii) from surgery to postoperative month 3. Categorical safety outcomes were summarized as n/N (%) with two-sided exact (Clopper–Pearson) 95% confidence intervals, and between-group comparisons were performed using Fisher’s exact test because of sparse events.

### Sample size calculation

The planned sample size was based on the primary endpoint. Assuming a moderate effect size (Cohen’s d ≈ 0.6) for between-group difference in cavity volume reduction at 3 months, with a two-sided *α* = 0.05 and statistical power (1–β) = 0.90, 48 patients per group were required. Allowing for an anticipated dropout rate of ~15%, the target enrollment was 57 patients per group, yielding a total of 114 patients. This sample size was deemed sufficient for a prospective observational comparison of two treatment strategies.

No interim analyses were planned.

### Data collection and follow-up

Clinical assessments, laboratory tests, imaging studies, and questionnaire-based evaluations were performed at predefined time points: baseline, postoperative weeks 2, and 3 months. Data were collected into electronic case report forms (eCRFs) by trained staff. Data integrity was ensured through standardized procedures, double-entry checks, automated query systems, and periodic monitoring.

### Statistical analysis

All analyses followed a predefined statistical analysis plan. The full analysis cohort included all enrolled patients who underwent surgery and had at least one post-baseline assessment, and participants were analyzed according to their initially chosen treatment strategy at baseline (strategy-based analysis). Because treatment allocation was preference-based and the study aimed to evaluate real-world effectiveness of the chosen management strategy, we maintained baseline strategy classification throughout follow-up; missing outcome data were addressed using multiple imputation. The per-protocol set (PPS) included patients without major protocol deviations and who completed planned follow-up assessments. The safety set comprised all patients who underwent surgery and, for the treatment-group cohort, at least one ultrasound-assisted therapy session.

Continuous variables were summarized as mean ± standard deviation or median (IQR), and categorical variables as frequencies and percentages. Baseline characteristics between cohorts were compared using Student’s t-test or Mann–Whitney U test for continuous variables, and χ^2^ or Fisher’s exact test for categorical variables.

For the primary endpoint, analysis of covariance (ANCOVA) was performed with combined-therapy group as the main factor, adjusting for baseline cavity volume and relevant covariates (e.g., age, BMI, baseline inflammatory indices) to mitigate confounding from preference-based group selection. Adjusted between-group differences were presented with 95% confidence intervals and *p* values.

To further address potential treatment-selection bias due to preference-based allocation, we performed propensity score (PS) weighting based on a prespecified confounder set reflecting demographics, comorbidity, baseline disease severity, and baseline inflammatory/nutritional/function status. Covariates included age, sex, BMI, comorbidity category, baseline residual cavity volume, baseline WBC, CRP, and PCT, baseline hemoglobin and albumin, baseline creatinine and ALT, and baseline ADL.

The PS (probability of choosing the combined-therapy strategy) was estimated using logistic regression. Given the modest sample size and to reduce sensitivity to extreme PS values, we applied overlap weighting (treated weight = 1—PS; control weight = PS) to target the average treatment effect in the overlap population. Covariate balance was assessed using standardized mean differences (SMDs), with |SMD| < 0.10 considered adequate. Overlap-weighted linear (for continuous outcomes) and logistic (for binary outcomes) regression models with robust (HC1) standard errors were used to estimate adjusted between-group differences (or odds ratios) and 95% confidence intervals; for outcomes with baseline measurements, the corresponding baseline value was additionally included in the outcome model to improve precision (doubly robust estimation).

Given two prespecified follow-up time points (2 weeks and 3 months), we performed timepoint-specific comparisons. For each continuous outcome at each follow-up, we fitted an ANCOVA model with combined-therapy group as the main factor, adjusting for the corresponding baseline value and prespecified covariates. Binary outcomes were compared at each time point using χ^2^/Fisher’s exact tests and, where appropriate, covariate-adjusted logistic regression. As a sensitivity analysis, we additionally fitted a linear mixed-effects model for repeated measures including fixed effects for group, time (2 weeks and 3 months), and group × time interaction, with a subject-level random intercept to account for within-subject correlation.

*Multiplicity control*. To address multiple comparisons across secondary endpoints, we controlled the false discovery rate (FDR) using the Benjamini–Hochberg (BH) procedure at q = 0.05. The BH adjustment was applied separately within each follow-up time point (postoperative week 2 and month 3). For each time point, the FDR family included the prespecified secondary outcomes assessed at that visit (e.g., inflammatory markers and laboratory indices, functional outcomes, and quality-of-life measures). The primary endpoint was analyzed using a prespecified model without multiplicity adjustment; for transparency, we report both nominal *p* values and BH-adjusted p values for secondary outcomes.

*Missing data and multiple imputation*. The extent and pattern of missing data were summarized by outcome and time point. Under a missing-at-random assumption, missing values were handled using multiple imputation by chained equations (MICE) to generate m = 20 imputed datasets, with 20 iterations per dataset. Continuous variables were imputed using predictive mean matching (±M); binary variables using logistic regression; nominal categorical variables using multinomial logistic regression; and ordinal variables using proportional odds models. The imputation model included treatment strategy group, all outcomes at all available time points, baseline values of each outcome (when available), and prespecified baseline covariates (e.g., age, sex, BMI, comorbidities, and baseline disease severity indicators). Additional auxiliary variables related to missingness and/or outcomes were also included when available to improve imputation performance. Estimates from each imputed dataset were analyzed using the prespecified models and pooled using Rubin’s rules. Convergence and plausibility were assessed by inspecting trace plots of selected imputed variables across iterations and comparing the distributions of observed versus imputed values.

## Results

1 Patient disposition

Between July 2023 and June 2025, 114 patients with chronic empyema were prospectively enrolled. After standardized clinical consultation regarding both treatment options, 57 patients elected to undergo free VLMCF surgery alone, and 57 chose additional ultrasound-mediated transdermal Qianjin Weijing Decoction. All included patients underwent surgery and were analyzed according to their initially chosen treatment strategy at baseline (strategy-based analysis). No planned crossover between strategies occurred. Follow-up completion exceeded 85% at all major assessment points. Missing observations were handled using multiple imputation.

2 Baseline characteristics

Baseline demographic and clinical variables were generally comparable between the two preference-based groups (Table 1). No significant differences were observed in age, sex, BMI, comorbidities, baseline CRP, PCT, albumin, creatinine, ALT, baseline cavity volume, or ADL score (all *p* > 0.05) (Tables 1, 2).

**Table 1:** Comparison of baseline levels of categorical variables between groups.

Variable	χ^2^	*p*_value	SMD
Sex	0.609422	0.435006	−0.18279
Underlying diseases	1.142857	0.76674	NA

**Table 2:** Comparison of baseline levels of continuous variables between groups.

Variable	Group 1 (mean±SD)	Group 2 (mean±SD)	*p*_value	SMD
Age	63.09 ± 9.33	62.93 ± 10.27	0.931667	−0.0161
BMI	21.31 ± 1.27	20.94 ± 1.23	0.120079	−0.29341
WBC_baseline (×10^9^/L)	6.95 ± 2.13	6.08 ± 2.32	0.038707	−0.39188
CRP_baseline (mg/L)	13.32 ± 11.35	12.53 ± 13.87	0.73852	−0.06269
PCT_baselinen (ng/mL)	0.07 ± 0.09	0.07 ± 0.08	0.773123	−0.05413
Hb_baseline (g/L)	122.84 ± 13.13	126.54 ± 13.96	0.147568	0.273164
Alb_baseline (g/L)	36.23 ± 2.81	36.06 ± 2.43	0.727568	−0.06542
Cr_baseline (μmol/L)	61.3 ± 11.53	62.32 ± 11.21	0.63388	0.089459
ALT_baseline (U/L)	21.91 ± 18.01	20.28 ± 13.55	0.585846	−0.10238
CavityVol_baseline (mL)	178.68 ± 66.83	179.48 ± 56.16	0.944894	0.012977
ADL_Basline	91.05 ± 12.2	87.81 ± 16.04	0.2267	−0.22779

Reference range institutional ULN for procalcitonin (PCT) = <0.05 ng/mL.

A modest baseline imbalance was noted for WBC levels (*p* = 0.0387; standardized mean difference [SMD] ≈ 0.39), and mild SMD deviations were also observed for BMI, hemoglobin, and ADL. These variables were therefore included as covariates in adjusted analyses to mitigate potential residual confounding. Taken together, although treatment selection was patient-driven rather than randomized, the two groups demonstrated acceptable baseline comparability and allowed for meaningful adjusted comparative analysis.

## Between-group comparisons based on ANCOVA analyses

### Between-group differences at 2 weeks after surgery

#### Primary outcomes at 2 weeks

**Figure 1:**
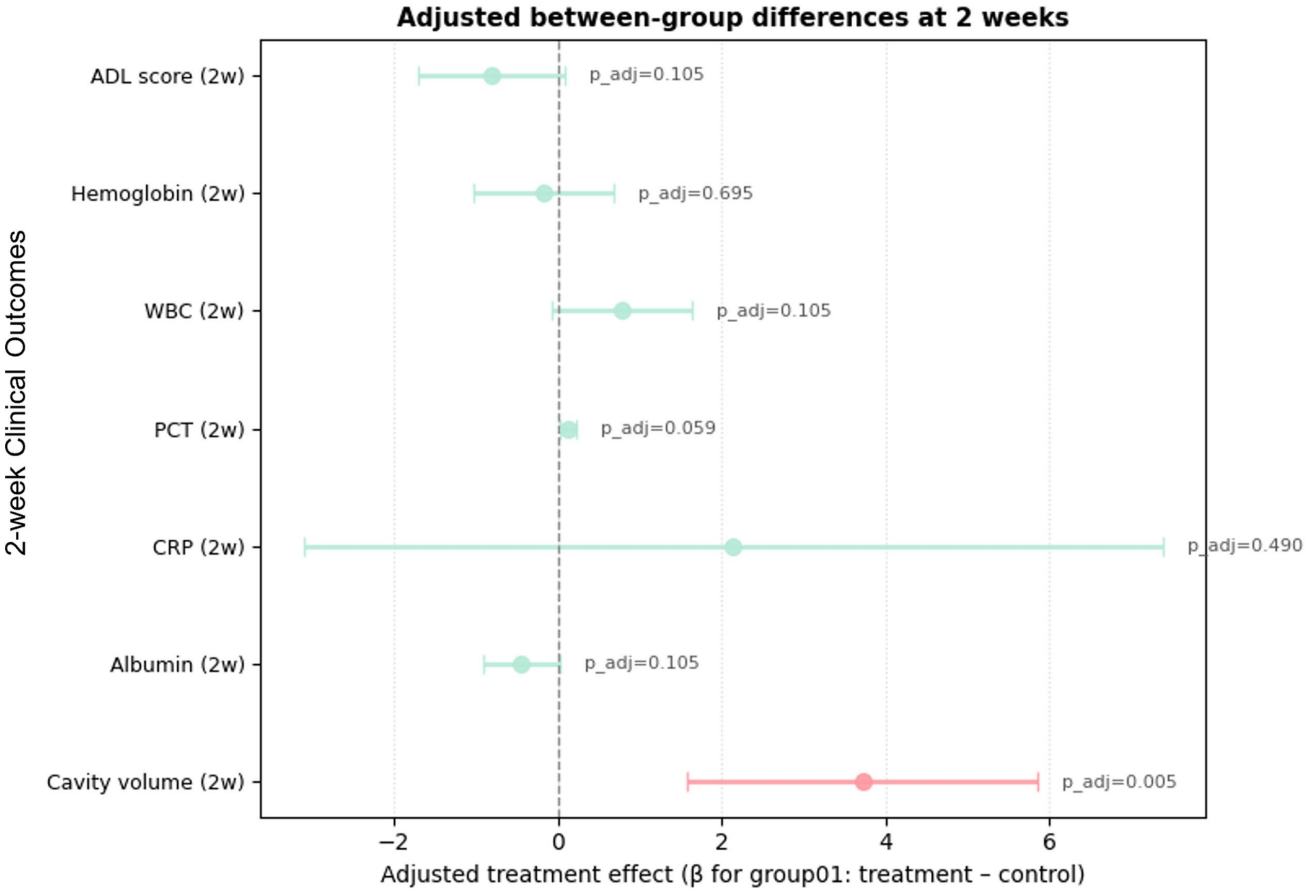
Forest plot of between-group multiple comparisons at 2 weeks postoperatively.

After adjustment for baseline cavity volume and mildly imbalanced covariates, the early treatment effect remained robust. At 2 weeks, the combined-therapy group (Group 1) had an adjusted mean 3.72 mL greater reduction in cavity volume than the surgery-alone group (Group 2) (95% CI 1.588–5.84; *p* = 0.00077; p_adj = 0.0054), indicating accelerated early cavity resolution (Figure 1; Table 3). Figure 1 displays the adjusted between-group differences from ANCOVA for the primary outcome and key secondary endpoints, with values to the left of zero favoring the combined-therapy group. While the combined regimen produced a significantly greater early reduction in cavity volume, most secondary endpoints showed only small, non-significant between-group differences at this time point.

**Table 3:** Between-group comparisons based on ANCOVA analyses. (Group 1–Group 2).

Outcome	Time	Beta/t/χ^2^	CI_low	CI_high	*p*	p_adj
CavityVol_2 W	2w	−3.72(−2.08%)	−5.8456116	−1.58840	0.000771	0.005398
ALB_2 W	2w	−0.44225	−0.91063	0.026125	0.063966	0.104955
CRP_2 W	2w	2.140629	−3.09921	7.380466	0.419849	0.489824
PCT_2 W	2w	0.119182	0.021857	0.216506	0.016864	0.059025
WBC_2w	2w	0.778383	−0.07447	1.631239	0.073224	0.104955
HB_2 W	2w	−0.17003	−1.02743	0.687373	0.695037	0.695037
ADL_2 W	2w	−0.81074	−1.70453	0.083038	0.074968	0.104955
SF36_2 W	2 W	t = 2.395			0.018	
Post_Comp_2 W	2 W	χ^2^ = 2.21			0.698	
CavityVol_3 m	3 m	−0.44(−0.25%)	−1.47681	0.604658	0.408063	0.700562
Alb_3 m	3 m	0.026941	−0.05204	0.105921	0.500401	0.700562
CRP_3M	3 m	3.381756	−0.20353	6.967038	0.064241	0.224843
PCT_3 m	3 m	0.10259	0.053324	0.151856	<0.0001	0.000507
WBC_3 m	3 m	0.336639	−0.20176	0.875041	0.217919	0.508478
Hb_3 m	3 m	−0.18446	−1.63113	1.262212	0.800949	0.934441
ADL_3 m	3 m	−0.02255	−0.81484	0.769735	0.955112	0.955112
SE36_3 m	3 m	−1.306			0.195	
Post_Comp_3 m	3 m	χ^2^ = 0.176			0.675	

#### Secondary outcomes at 2 weeks

At 2 weeks, adjusted analyses revealed no significant between-group differences in WBC or CRP and only small, non-significant trends in PCT, indicating broadly comparable early inflammatory control. Nutritional indices (albumin and hemoglobin) showed similar early recovery in both groups, with no adjusted contrasts remaining significant after false discovery rate correction. ADL scores improved substantially from baseline in both cohorts, reflecting marked early postoperative recovery. In contrast, SF-36 demonstrated a transient quality-of-life advantage in the combined-therapy group, which exhibited higher SF-36 scores than the surgery-alone group, consistent with faster early symptom relief and improved perceived wellbeing.

### Between-group differences at 3 months after surgery

#### Primary outcome at 3 months

**Figure 2:**
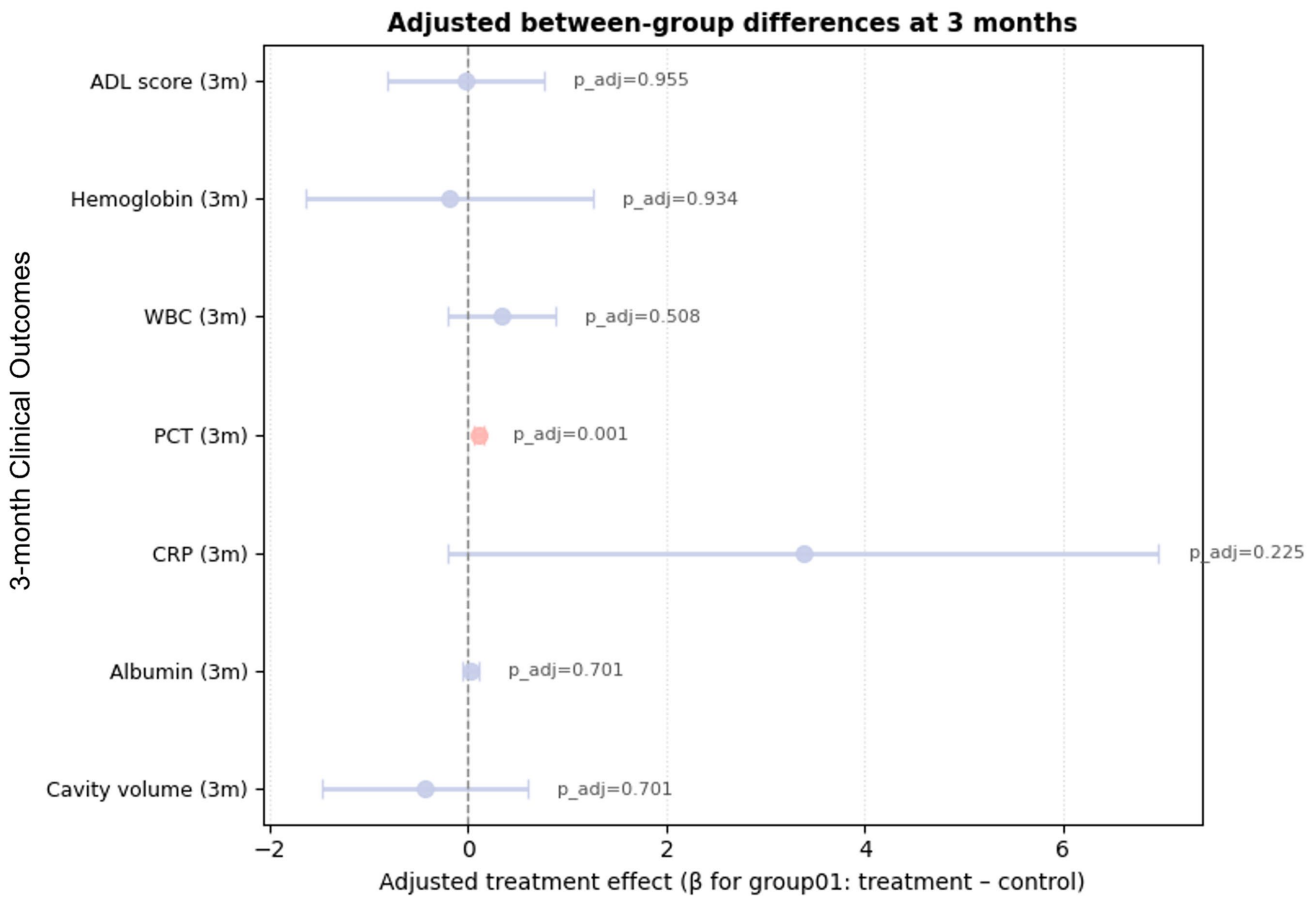
Forest plot of between-group multiple comparisons at 3 months postoperatively.

At 3 months, the adjusted between-group difference in residual cavity volume was small and non-significant (*β* = −0.44 mL; 95% CI − 1.48 to 0.60; FDR-adjusted *p* = 0.70), indicating that cavity volumes had largely converged and the early advantage of the combined regimen was no longer apparent (Figure 2; Table 3). Figure 2 summarizes the adjusted ANCOVA effects for the primary outcome and key secondary endpoints; most confidence intervals cross the null line, consistent with attenuation of the early treatment benefit and overall comparable clinical profiles between groups by 3 months.

#### Secondary outcomes at 3 months

By 3 months, inflammatory and nutritional markers were largely similar between treatment strategies. Adjusted WBC and CRP values remained comparable, whereas PCT showed the clearest distinction: the combined-therapy group had a significantly higher adjusted PCT level (adjusted mean difference 0.10; 95% CI 0.05 to 0.15; FDR-adjusted *p* = 0.0005). Absolute PCT values at baseline, 2 weeks, and 3 months for both groups are provided in Supplementary Table S4, together with the institutional ULN (<0.05 ng/mL), to facilitate interpretation of the clinical magnitude of between-group differences. In contrast, albumin and hemoglobin trajectories were broadly comparable, with all adjusted contrasts remaining non-significant after multiple-testing correction, indicating no meaningful effect on medium-term nutritional reconstitution. ADL scores remained high in both cohorts without clinically important between-group differences, and SF-36 scores had converged, with no residual quality-of-life advantage, implying that the early SF-36 benefit in the combined-therapy group did not persist at 3 months.

**Figure 3:**
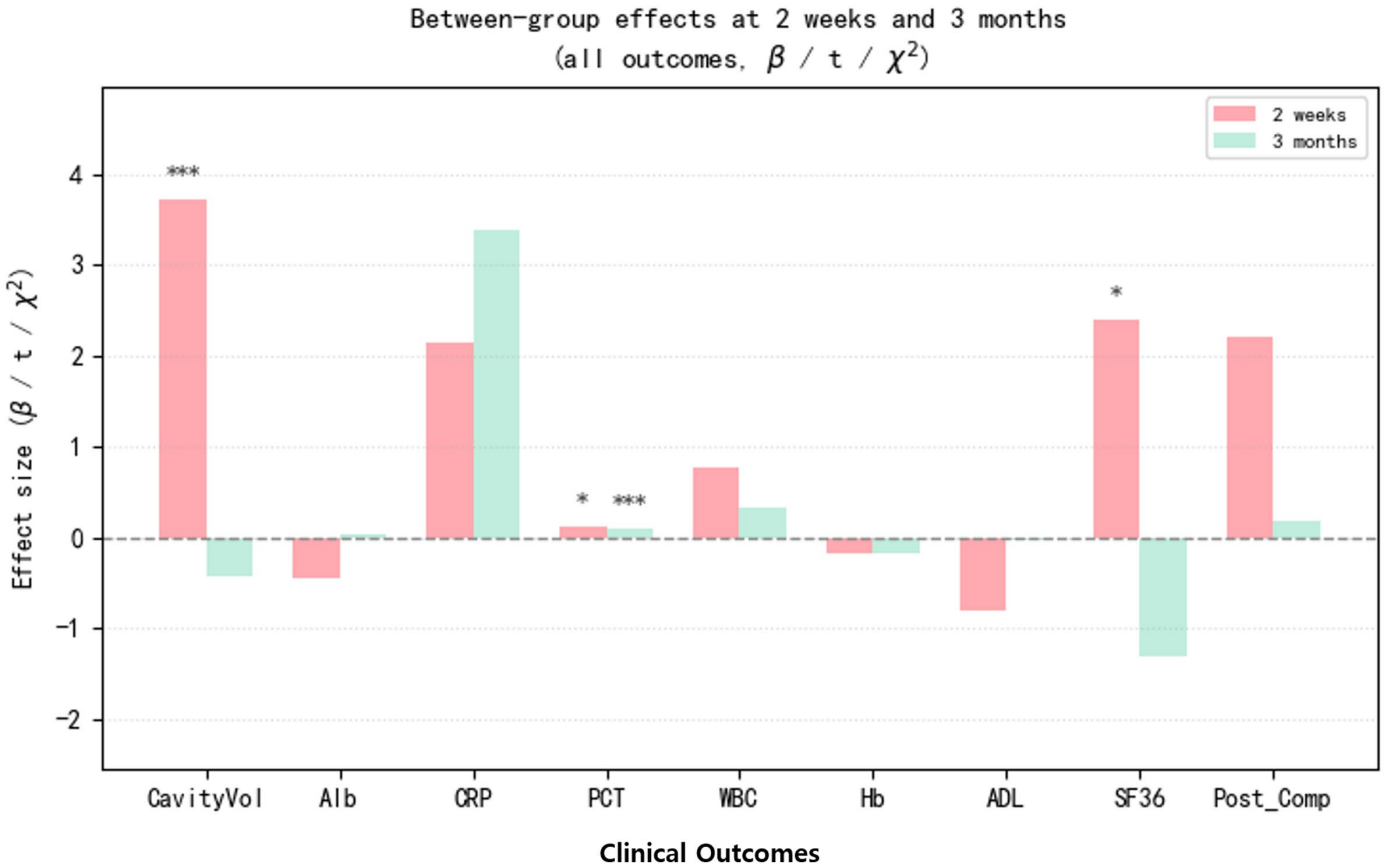
Bar plot of between-group effects at 2 weeks and 3 months (all outcomes, *β*/*t*/χ^2^).

Figure 3 displays the adjusted between-group effects for all primary and secondary outcomes at 2 weeks and 3 months after surgery, expressed as effect sizes (*β*, t, or χ^2^; combined therapy minus surgery alone). Pink bars represent estimates at 2 weeks and green bars those at 3 months, with asterisks indicating statistically significant differences.

At 2 weeks, the combined-therapy group showed a clearly larger reduction in residual cavity volume (CavityVol) and a transient advantage in quality of life (SF-36), with smaller but still favorable effects on procalcitonin (PCT). By 3 months, most outcomes had converged between groups and effect sizes were small and non-significant, except for PCT, which remained significantly lower in the combined-therapy group, suggesting more sustained control of infection-related inflammation.

### Safety

No treatment-related serious adverse events occurred in either group during follow-up. Postoperative surgical complications were uncommon in both groups. At postoperative week 2, any complication occurred in 5/57 (8.8%) patients in the combined-therapy group and 4/57 (7.0%) patients in the surgery-alone group (Fisher’s exact *p* = 1.000). By month 3, any complication occurred in 2/57 (3.5%) vs. 4/57 (7.0%), respectively (*p* = 0.679). Detailed event categories with exact 95% CIs are provided in Table 4.

**Table 4:** Postoperative surgical complications by combined-therapy group (2 weeks and 3 months).

Event		Combined therapy (Group 1) n/N (%) [95%CI]	Surgery alone (Group 2) n/N (%) [95%CI]	P (Fisher)
2-weeks	Any postoperative complication	5/57 (8.8%) [2.9–19.3]	4/57 (7.0%) [1.9–17.0]	1
	Partial flap necrosis	3/57 (5.3%) [1.1–14.6]	2/57 (3.5%) [0.4–12.1]	1
	Wound/flap infection	0/57 (0.0%) [0.0–6.3]	1/57 (1.8%) [0.0–9.4]	1
	Bleeding/hematoma	1/57 (1.8%) [0.0–9.4]	1/57 (1.8%) [0.0–9.4]	1
	Respiratory failure	1/57 (1.8%) [0.0–9.4]	0/57 (0.0%) [0.0–6.3]	1
3-months	Any postoperative complication	2/57 (3.5%) [0.4–12.1]	4/57 (7.0%) [1.9–17.0]	0.679
	Flap atrophy/ulcer/infection	2/57 (3.5%) [0.4–12.1]	4/57 (7.0%) [1.9–17.0]	0.679
	Pleural complications / recurrent BPF	0/57 (0.0%) [0.0–6.3]	0/57 (0.0%) [0.0–6.3]	1
	Chest-wall deformity/asymmetry	0/57 (0.0%) [0.0–6.3]	0/57 (0.0%) [0.0–6.3]	1

No treatment-emergent AEs or SAEs were observed (Supplementary Table S1).

### Sensitivity analyses

#### Propensity score-based sensitivity analyses

As an additional sensitivity analysis to mitigate measured confounding, PS overlap-weighted analyses achieved excellent balance across all prespecified covariates (maximum |SMD| reduced from 0.392 unweighted to 0.053 after weighting; Supplementary Figure S1). The overlap-weighted estimates were consistent with the primary ANCOVA results for the primary endpoint at 2 weeks (adjusted mean difference [treatment - control] -3.73 mL; 95% CI –5.80 to −1.66; *p* = 0.0006) and for procalcitonin at 3 months (−0.107 ng/mL; 95% CI –0.152 to −0.061; *p* < 0.0001), while cavity volume at 3 months remained not significantly different between groups (0.46 mL; 95% CI –0.63 to 1.54; *p* = 0.41). Full PS-weighted results are provided in Supplementary Table S2.

To assess the robustness of our findings to missing-data handling, we compared complete-case ANCOVA results with those obtained after multiple imputation (MI). For each continuous outcome at 2 weeks and 3 months, identical ANCOVA models were fitted in the complete-case dataset and in the Rubin-pooled MI dataset, with combined-therapy group as the main factor and adjustment for the corresponding baseline value and key prognostic covariates (age, BMI, baseline ADL). The adjusted group effect (*β* for group01) and its 95% confidence interval and *p*-value were summarized side by side for complete-case and MI-pooled analyses.

**Figure 4:**
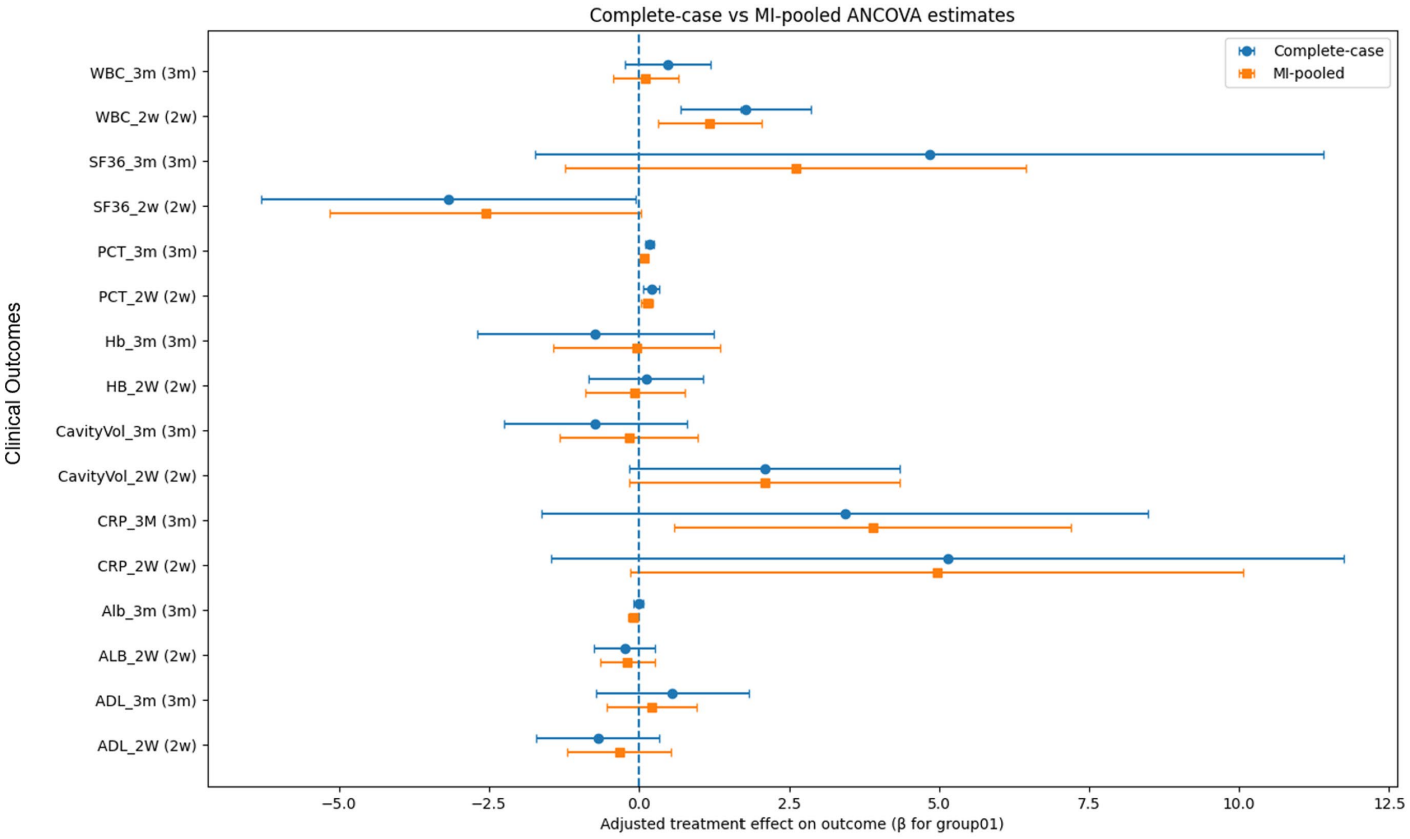
Forest plot of sensitivity analyses based on multiple imputation.

Across all prespecified endpoints, the direction of the treatment effect was identical in complete-case and MI-based models, and the magnitudes of the β-coefficients were very similar. Outcomes that were statistically significant in the complete-case analysis (e.g., early reduction in cavity volume and PCT at follow-up) remained significant after MI, whereas outcomes that were non-significant also remained non-significant. No endpoint changed its qualitative inference (significant vs. non-significant) when moving from complete-case to MI-pooled results, indicating that the primary conclusions were robust to the choice of missing-data strategy (Figure 4).

#### Missing data

Missingness by outcome and time point is summarized in Supplementary Table S3. The primary endpoint (cavity volume) had complete baseline data, with missingness of 7.9% at postoperative week 2 and 37.7% at month 3. Follow-up laboratory measures showed greater missingness at month 3 (approximately 41–42% for WBC, CRP, PCT, hemoglobin, and albumin), whereas missingness at week 2 was lower for most laboratory outcomes. Functional outcomes also had incomplete follow-up (ADL missingness 34.2% at week 2 and 46.5% at month 3), and SF-36 had missingness of 17.5% at week 2 and 42.1% at month 3. Postoperative complications were fully recorded at week 2 (0% missing), but had 24.6% missingness at month 3. As prespecified, missing outcomes were handled using multiple imputation, and complete-case analyses were performed as sensitivity analyses.

#### Imputation diagnostics

We evaluated the plausibility of the imputed values by comparing pre- and post-imputation distributions for key continuous variables, including cavity volume, CRP, PCT, ADL, and SF-36 at 2 weeks and 3 months. For each variable, we compared means and standard deviations from the observed data with those from the MI-pooled dataset, and examined MI values in patients with observed versus imputed measurements. Post-imputation means and standard deviations closely matched those of the complete cases, and the distributions of imputed values were similar to those of the observed values, without evidence of systematic shifts or artificial compression.

Taken together, the close agreement between complete-case and MI-based ANCOVA estimates and the stable pre−/post-imputation distributions indicate that the MI procedure preserved the underlying data structure and did not introduce material bias, supporting the robustness and credibility of the primary efficacy analyses.

To further evaluate the potential for systematic bias due to attrition, we compared the baseline characteristics of participants who completed the 3-month follow-up for the primary outcome (*N* = 71) with those who did not (*N* = 43). As shown in Supplementary Table S5, there were no significant differences between completers and non-completers regarding baseline disease severity (e.g., initial cavity volume, *p* = 0.21; PCT, *p* = 0.60), nutritional status (Albumin, *p* = 0.72), or functional baseline (ADL, *p* = 0.59). While a higher proportion of males was noted in the non-completer group (79.1% vs. 54.9%, *p* = 0.016), likely reflecting earlier return to work or geographic mobility, key clinical prognostic factors remained balanced. These findings support the missing-at-random (MAR) assumption, indicating that attrition was not primarily driven by the severity of the empyema at baseline.

### Summary

**Figure 5:**
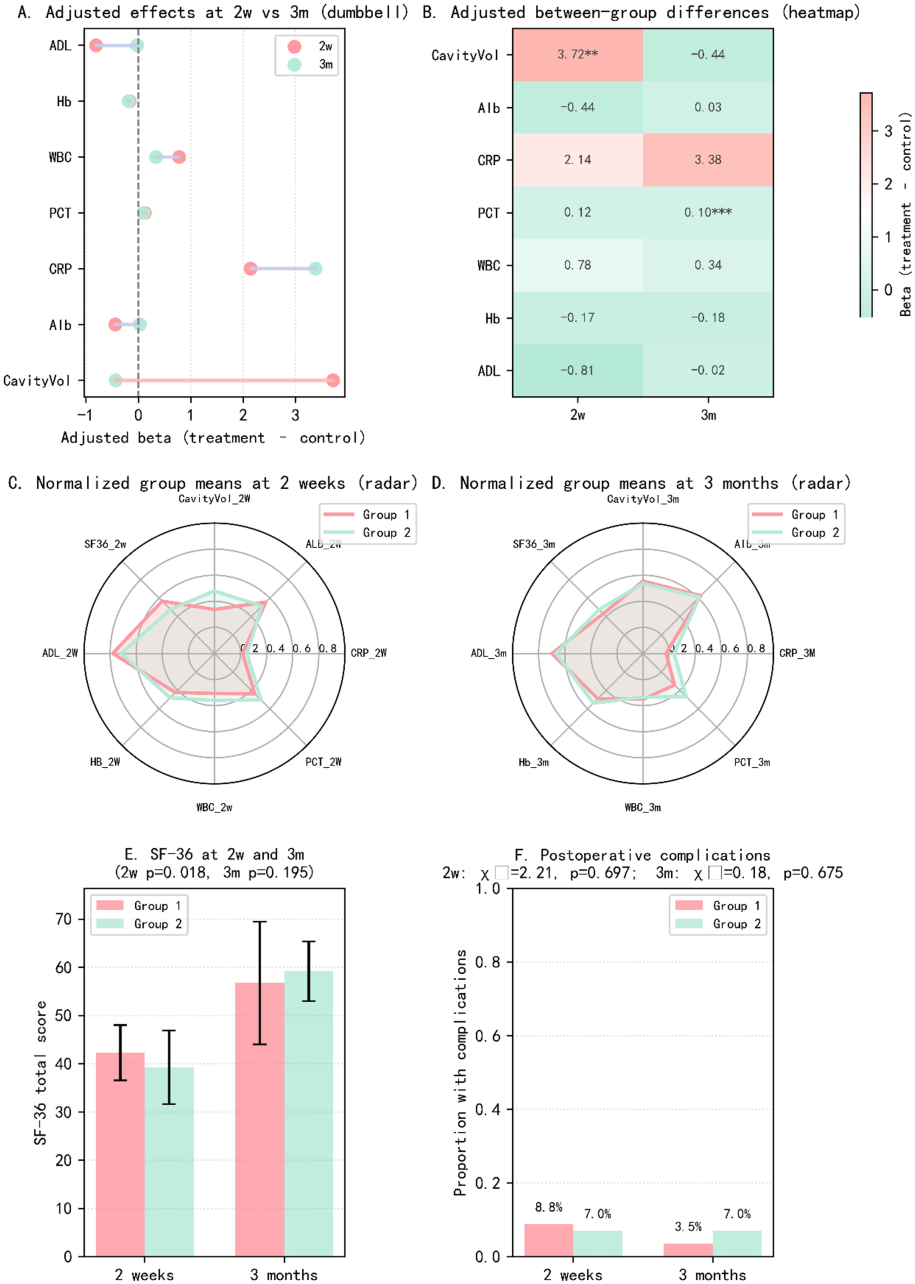
Multidimensional visualization of between-group effects at 2 weeks and 3 months. **(A)** Adjusted effects at 2 weeks vs. 3 months (dumbbell plot). Each line shows the adjusted between-group difference (beta, combined therapy minus surgery alone) for a given outcome at 2 weeks (pink) and 3 months (green), with dots representing point estimates from ANCOVA models and the vertical dashed line indicating no effect. Values to the left of zero favor the combined-therapy group. **(B)** Adjusted between-group differences (heatmap). Heatmap of adjusted betas at 2 weeks and 3 months for all continuous outcomes. Cells are color-coded by effect size (green for lower values, red for higher values in the combined-therapy group), with asterisks denoting FDR-adjusted statistical significance (*p < 0.05, **p < 0.01, ***p < 0.001). **(C)** Normalized group means at 2 weeks (radar plot). Radar chart of normalized group means for cavity volume, nutritional markers, inflammatory biomarkers, ADL, and SF-36 at 2 weeks, comparing the combined-therapy group (Group 1) with the surgery-alone group (Group 2). **(D)** Normalized group means at 3 months (radar plot). Radar chart of the same outcomes at 3 months, illustrating convergence of most clinical parameters between groups over time. **(E)** SF-36 total scores at 2 weeks and 3 months. Bar graph of SF-36 total scores (mean ± SD) by group and time point. At 2 weeks, the combined-therapy group shows higher SF-36 scores (*p* = 0.018), indicating better early health-related quality of life; by 3 months, scores are similar between groups (*p* = 0.195). **(F)** Postoperative complications at 2 weeks and 3 months. Proportion of patients experiencing postoperative complications in each group at 2 weeks and 3 months. The overall complication rates are low and do not differ significantly between the combined-therapy and surgery-alone groups (2 weeks: χ^2^ = 2.21, *p* ≈ 0.70; 3 months: χ^2^ = 0.18, *p* ≈ 0.68).

**Figure 6:**
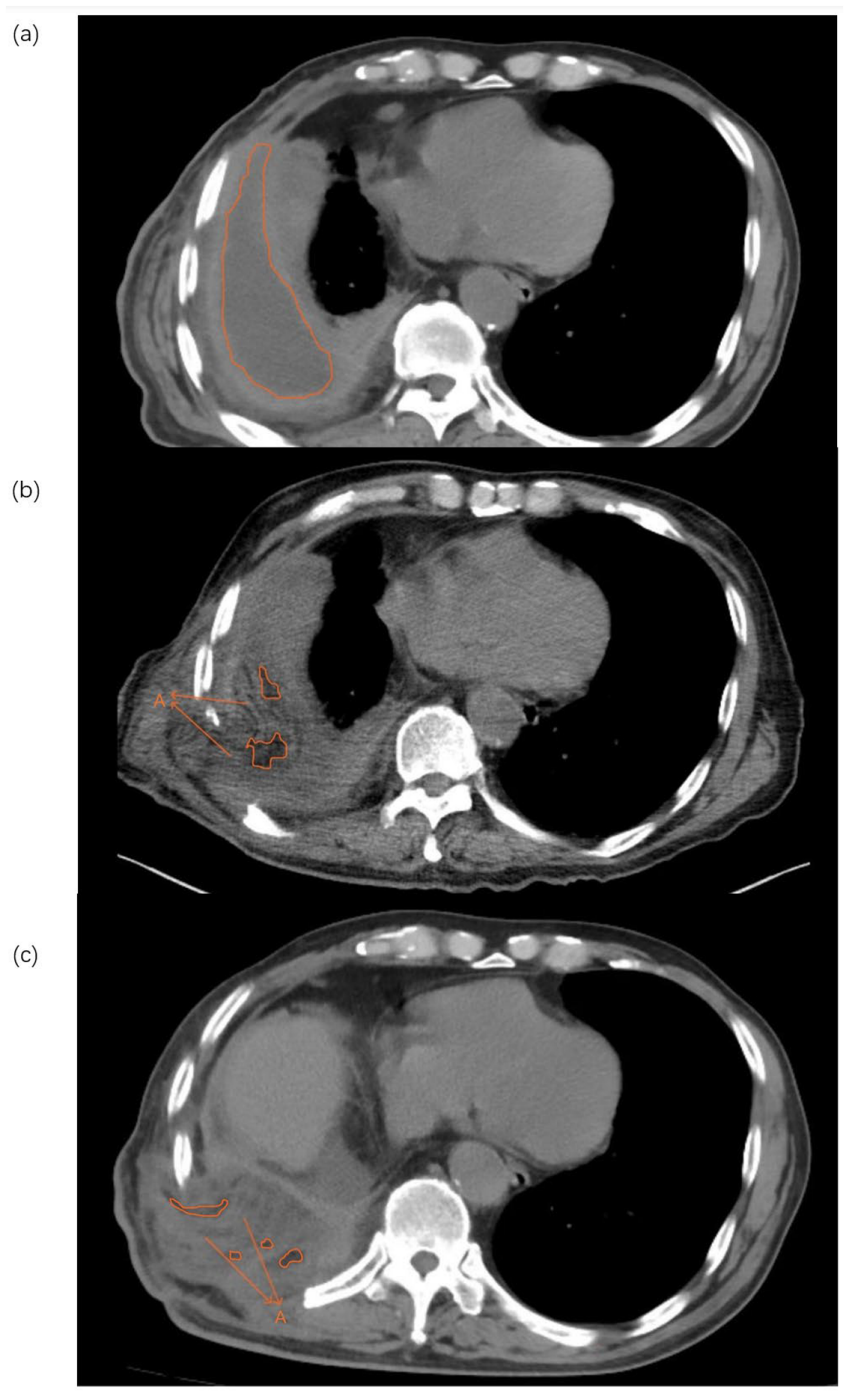
Sequential axial CT images with segmentation overlays and CT-derived cavity volumes of a representative patient in the combined-therapy group (Group 1). **(a)** Preoperative baseline: A large empyema cavity is outlined by the orange contour; the total cavity volume, calculated by 3D segmentation/volumetric analysis of the entire thin-slice CT dataset, was 1164.88 mL. **(b)** 2 weeks postoperatively: The residual cavity (orange contour) decreased to 22.10 mL. The arrow indicates the transplanted free vastus lateralis musculocutaneous flap occupying/obliterating the former empyema space. **(c)** 3 months postoperatively: The flap remains stable and well filled, with a small residual cavity (orange contour); the CT-derived residual cavity volume further decreased to 12.32 mL. Volumes were computed from 3D segmentation/volumetry of the full CT series; the displayed axial slices are illustrative.

Taken together, Figure 5 and Table 5 shows that the combined regimen yields a clearly greater early reduction in residual cavity volume and a transient improvement in SF-36 scores at 2 weeks, while most inflammatory, nutritional, and functional outcomes differ only modestly between groups. Figure 6 provides a representative visual example of the imaging assessment, illustrating the marked early shrinkage of the empyema cavity on sequential CT (with segmentation overlays) and the corresponding CT-derived cavity volumes after surgery in the combined-therapy group. By 3 months, clinical profiles—including cavity volume, quality of life, and complication rates—have largely converged, with the exception of more favorable procalcitonin profiles in the combined-therapy group reported in the main text.

**Table 5:** Summary of main effects.

Outcome	2 weeks	3 months
Residual cavity volume	Significantly better in combined group	Similar between groups
PCT	Trend toward improvement	Significantly better in combined group
CRP, WBC, Albumin, ADL	No meaningful differences	No meaningful differences
SF-36	Significantly better in combined group	No meaningful differences
Safety	Comparable	Comparable

## Discussion

In this prospective, preference-based cohort of patients with chronic empyema undergoing free vastus lateralis/anterolateral thigh musculocutaneous flap reconstruction, adjunctive ultrasound-assisted transdermal application of Qianjin Weijing decoction was associated with a slightly greater early reduction in residual cavity volume and a transient improvement in SF-36 at 2 weeks, while most nutritional, inflammatory, functional, and safety outcomes were broadly similar to flap surgery alone. By 3 months, cavity volumes and health-related quality of life measures largely converged between groups, and any between-group differences were small and should be interpreted cautiously. Overall, the data suggest that any potential advantage of the integrative regimen—if present—may be confined mainly to the early postoperative period rather than reflecting a sustained mid-term structural difference.

At 2 weeks, the adjusted between-group difference in residual cavity volume was statistically significant (*β* = −3.72 mL; 95% CI − 5.85 to −1.59), but the absolute magnitude was modest relative to baseline cavity size (baseline mean ≈179 mL). This corresponds to an adjusted difference of approximately −2.08% of baseline (95% CI −3.26 to −0.89%). At 3 months, the adjusted difference was smaller and not statistically significant (*β* = −0.44 mL; 95% CI − 1.48 to 0.60), corresponding to approximately −0.25% of baseline (95% CI − 0.82 to 0.34%). Overall, the imaging effect size appears small, and its clinical importance depends on whether it aligns with patient-important outcomes. Because chest drainage duration, length of hospital stay, and reintervention were not prespecified or systematically collected in this study, whether the early imaging difference translates into tangible clinical benefit remains uncertain and should be evaluated in future trials.

Our findings align with prior evidence that vascularized muscle or musculocutaneous flaps can obliterate chronic empyema cavities and contribute to source control in advanced cases, particularly when residual spaces are large or complex (13). Thoracomyoplasty with well-vascularized tissue is widely regarded as an effective option for refractory empyema, and free vastus lateralis/anterolateral thigh flaps are often selected for their bulk, reliability, and perfusion characteristics (14–16). Nevertheless, postoperative cavity involution may be gradual in some patients, and adjunctive, low-burden rehabilitation strategies remain of interest for supporting early recovery.

Qianjin Weijing decoction has historically been used for suppurative pulmonary conditions, and studies in other respiratory inflammatory/infectious settings suggest that Weijing-based formulations may have anti-inflammatory and mucus-regulating properties (10, 17). As highlighted in recent cutting-edge research regarding respiratory microenvironments (18), the precise modulation of the localized inflammatory niche is critical for resolving chronic suppurative conditions. Our findings align with this principle, suggesting that the ultrasound-mediated delivery of *Qianjin Weijing* decoction optimizes the local environment within the empyema cavity, thereby facilitating rapid tissue remodeling and space obliteration observed in the early postoperative phase. Ultrasound-mediated transdermal drug delivery (sonophoresis/phonophoresis) can enhance skin permeability and local delivery of topical agents, providing a non-invasive route that may reduce the need for additional systemic administration (7). On this basis, ultrasound-assisted transdermal application of the study herbal solution was evaluated as an adjunct to standard postoperative care. Given the observational, preference-based design, the results should be interpreted as adjusted associations rather than causal effects, and residual confounding by unmeasured factors cannot be excluded (19).

The isolated procalcitonin (PCT) difference at 3 months warrants conservative interpretation. By that time point, most clinical and laboratory outcomes had largely converged between groups, and an isolated biochemical signal—especially of small absolute magnitude—should not be interpreted as sustained infection control in the absence of corroborating clinical differences. As shown in Supplementary Table S4, absolute PCT values at follow-up remained above the institutional upper limit of normal (ULN < 0.05 ng/mL) in both groups, suggesting that the between-group difference reflects a shift within an elevated range rather than normalization. Therefore, the 3-month PCT finding is best considered exploratory and hypothesis-generating, and it should be corroborated by patient-important clinical endpoints and longer follow-up in future studies (5).

The transient SF-36 improvement at 2 weeks may be relevant to early recovery; however, it should be interpreted cautiously in a non-blinded, preference-driven setting. Participants who opted for integrative therapy may have higher expectations and greater engagement with postoperative care, which can influence patient-reported outcomes independent of physiological effects (expectation/placebo and reporting effects). Therefore, the early SF-36 difference is best viewed as a short-term, patient-reported signal that requires confirmation in randomized or better-controlled designs using blinded outcome assessment where feasible.

Methodological strengths include standardized surgery, CT-based volumetry to quantify residual cavity volume, and prespecified analytic approaches to mitigate bias (covariate adjustment, overlap weighting, and multiple imputation for missing data). Three-dimensional CT reconstruction provides a more precise assessment than simple radiographic estimates and has been recommended in contemporary empyema surgery literature (16). Nevertheless, the primary limitation is structural: treatment allocation was based on patient preference rather than randomization, so residual confounding by unmeasured factors (e.g., motivation, engagement, health behaviors) is likely despite adjustment (19). Missing follow-up data—particularly at 3 months—also constrain inference. Multiple imputation was applied under a missing-at-random (MAR) assumption; however, MAR is not directly verifiable in a preference-based cohort, and differential follow-up related to recovery or engagement could introduce bias. Baseline characteristics were compared between participants with and without 3-month follow-up data (Supplementary Table S5), and complete-case analyses were performed as sensitivity analyses to assess robustness to missingness (Figure 4). Although attrition was substantial for several endpoints at month 3, the completer versus non-completer comparison showed broadly similar baseline clinical and physiological profiles, supporting the plausibility of the MAR assumption used for imputation. In addition, because SF-36 is a patient-reported outcome and the study was non-blinded with preference-based allocation, expectation and reporting effects may have contributed to the early SF-36 difference. Taken together, these considerations suggest that attrition is unlikely to have materially altered the main inferences regarding early postoperative cavity-volume differences, while the non-randomized, preference-based design remains the dominant constraint on causal interpretation.

In summary, adjunctive ultrasound-assisted transdermal application of Qianjin Weijing decoction was associated with small early differences in cavity volume and transient quality-of-life improvement without obvious safety penalties, but the observational, preference-based design precludes causal conclusions. Future randomized trials with longer follow-up and prespecified clinically meaningful endpoints (including chest drainage duration, length of stay, reintervention, and microbiological clearance), alongside mechanistic measures, are required to clarify whether these early associations translate into tangible clinical benefit and to establish optimal treatment parameters.

## Data Availability

The datasets presented in this study can be found in online repositories. The names of the repository/repositories and accession number(s) can be found at: 10.5281/zenodo.17914736.
