# Supplementary material for: Effectiveness of free vastus lateralis musculocutaneous flap transplantation plus ultrasound-mediated transdermal Qianjin Weijing decoction for chronic empyema: a prospective, preference-based observational study
Source: Front Med (Lausanne). 2026 Mar 12;13:1780744. doi: 10.3389/fmed.2026.1780744 (PMC13017900; doi:10.3389/fmed.2026.1780744)
Supplement: Supplementary file 1 [file Data_Sheet_1.docx]

**Supplementary material**

Table S1. Treatment-emergent AEs/SAEs table

| Event | Combined therapy (n=57) n/N (%) | Surgery alone (n=57) n/N (%) | P (Fisher) |
| --- | --- | --- | --- |
| Any AE potentially related to adjunct therapy | 0/57 (0.0%) [0.0–6.3] | 0/57 (0.0%) [0.0–6.3] | 1.000 |
| Erythema | 0/57 (0.0%) [0.0–6.3] | 0/57 (0.0%) [0.0–6.3] | 1.000 |
| Burning pain | 0/57 (0.0%) [0.0–6.3] | 0/57 (0.0%) [0.0–6.3] | 1.000 |
| Blistering | 0/57 (0.0%) [0.0–6.3] | 0/57 (0.0%) [0.0–6.3] | 1.000 |
| Palpitations | 0/57 (0.0%) [0.0–6.3] | 0/57 (0.0%) [0.0–6.3] | 1.000 |
| Chest tightness | 0/57 (0.0%) [0.0–6.3] | 0/57 (0.0%) [0.0–6.3] | 1.000 |
| Any SAE (all-cause) | 0/57 (0.0%) [0.0–6.3] | 0/57 (0.0%) [0.0–6.3] | 1.000 |

Values are n/N (%). Brackets show two-sided exact (Clopper–Pearson) 95% CIs. Fisher’s exact test was used for between-group comparisons due to sparse events. The AE observation window was [POD2–POD15 / throughout follow-up to 3 months] (specify).

Table S2. Propensity score overlap-weighted estimates for primary and secondary outcomes.

| Outcome | Effect type | Estimate (95% CI) | p | Baseline adjustment |
| --- | --- | --- | --- | --- |
| Residual cavity volume at 2 weeks | Adjusted mean difference | -3.728 (-5.799 to -1.656) | 0.0006 | baseline value |
| Albumin at 2 weeks | Adjusted mean difference | 0.409 (-0.088 to 0.906) | 0.1096 | baseline value |
| CRP at 2 weeks | Adjusted mean difference | -2.217 (-7.985 to 3.552) | 0.4530 | baseline value |
| Procalcitonin at 2 weeks | Adjusted mean difference | -0.131 (-0.223 to -0.040) | 0.0059 | baseline value |
| WBC at 2 weeks | Adjusted mean difference | -0.690 (-1.508 to 0.128) | 0.1011 | baseline value |
| Hemoglobin at 2 weeks | Adjusted mean difference | 0.199 (-0.668 to 1.065) | 0.6541 | baseline value |
| ADL at 2 weeks | Adjusted mean difference | 0.883 (0.045 to 1.721) | 0.0412 | baseline value |
| SF-36 at 2 weeks | Adjusted mean difference | 3.406 (0.629 to 6.183) | 0.0179 | none |
| Residual cavity volume at 3 months | Adjusted mean difference | 0.455 (-0.631 to 1.541) | 0.4132 | baseline value |
| Albumin at 3 months | Adjusted mean difference | -0.005 (-0.089 to 0.080) | 0.9127 | baseline value |
| CRP at 3 months | Adjusted mean difference | -3.599 (-7.187 to -0.011) | 0.0518 | baseline value |
| Procalcitonin at 3 months | Adjusted mean difference | -0.107 (-0.152 to -0.061) | <0.0001 | baseline value |
| WBC at 3 months | Adjusted mean difference | -0.406 (-0.931 to 0.120) | 0.1329 | baseline value |
| Hemoglobin at 3 months | Adjusted mean difference | 0.395 (-1.035 to 1.826) | 0.5891 | baseline value |
| ADL at 3 months | Adjusted mean difference | 0.095 (-0.608 to 0.799) | 0.7912 | baseline value |
| SF-36 at 3 months | Adjusted mean difference | -3.303 (-7.177 to 0.571) | 0.0975 | none |
| Postoperative complications at 2 weeks (any) | OR | 1.465 (0.194 to 11.036) | 0.7112 | none |
| Postoperative complications at 3 months (any) | OR | 0.468 (0.033 to 6.652) | 0.5746 | none |


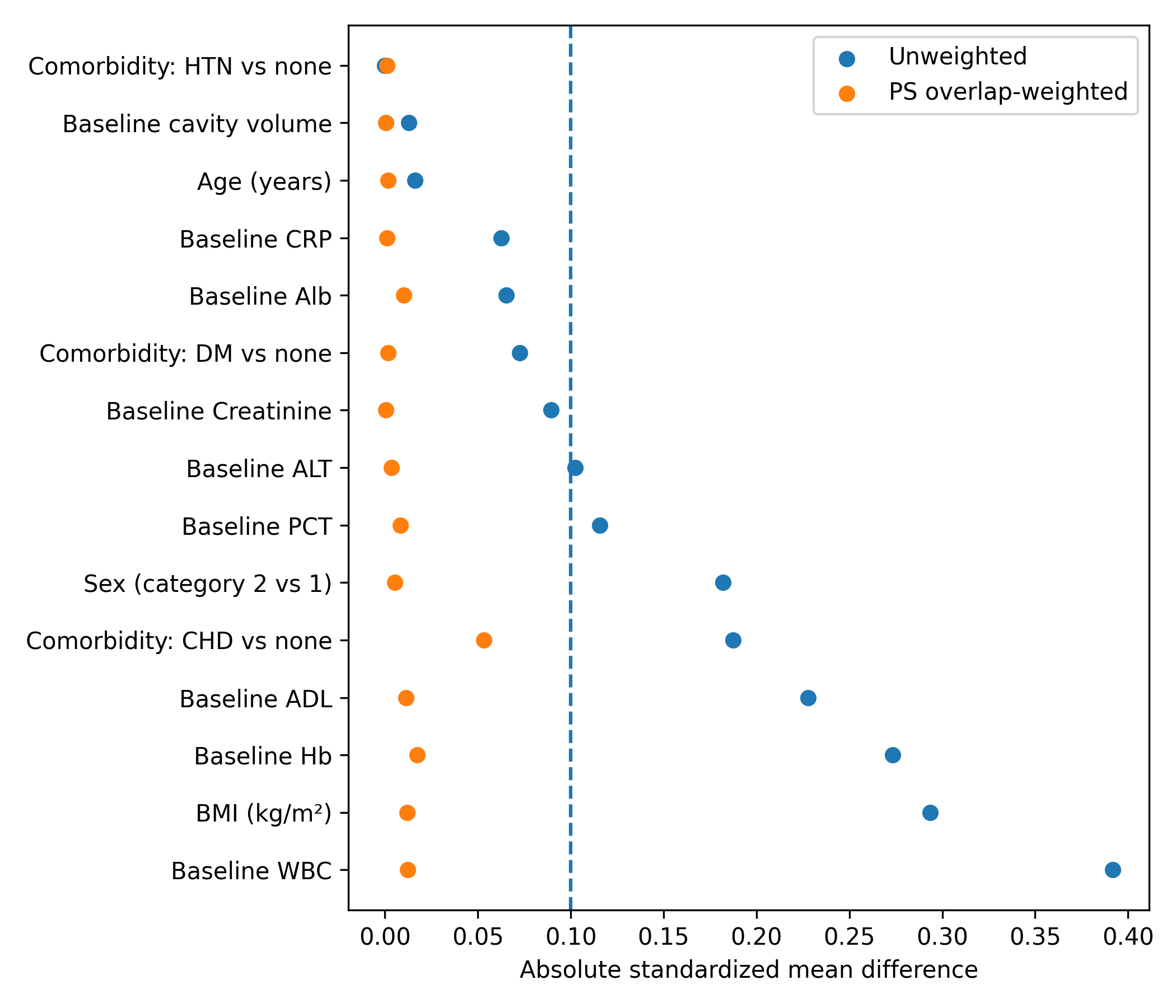


Figure S1. Covariate balance before and after propensity score overlap weighting (Love plot).

Table S3. Missingness from raw data

| Outcome | Baseline | Week 2 | Month 3 |
| --- | --- | --- | --- |
| Cavity volume (mL) | 0/114 (0.0%) | 9/114 (7.9%) | 43/114 (37.7%) |
| WBC (×10^9/L) | 0/114 (0.0%) | 27/114 (23.7%) | 47/114 (41.2%) |
| CRP (mg/L) | 0/114 (0.0%) | 28/114 (24.6%) | 48/114 (42.1%) |
| PCT (ng/mL) | 13/114 (11.4%) | 37/114 (32.5%) | 47/114 (41.2%) |
| Hemoglobin, Hb (g/L) | 0/114 (0.0%) | 20/114 (17.5%) | 47/114 (41.2%) |
| Albumin, Alb (g/L) | 0/114 (0.0%) | 28/114 (24.6%) | 47/114 (41.2%) |
| ADL score (points) | 0/114 (0.0%) | 39/114 (34.2%) | 53/114 (46.5%) |
| SF-36 (score) | — | 20/114 (17.5%) | 48/114 (42.1%) |
| Postoperative complications (coded) | — | 0/114 (0.0%) | 28/114 (24.6%) |

Table S4. Absolute procalcitonin (PCT) concentrations

| **Time** | **Group** | **n** | **Mean±SD (ng/mL)** | | **Median (IQR) (ng/mL)** | | **Within ULN <0.05** | |
| --- | --- | --- | --- | --- | --- | --- | --- | --- |
| Baseline | Group 1 | 57 | 0.079±0.086 | | 0.037 (0.025–0.100) | | 36/57 (63.2%) | |
| Baseline | Group 2 | 57 | 0.069±0.079 | | 0.037 (0.027–0.074) | | 42/57 (73.7%) | |
| 2 weeks | Group 1 | 57 | 0.859±0.249 | | 0.801 (0.720–0.931) | | 0/57 (0.0%) | |
| 2 weeks | Group 2 | 57 | 0.982±0.262 | | 0.970 (0.870–1.080) | | 0/57 (0.0%) | |
| 3 months | Group 1 | 57 | 0.763±0.138 | | 0.754 (0.675–0.845) | | 0/57 (0.0%) | |
| 3 months | Group 2 | 57 | 0.868±0.115 | | 0.855 (0.813–0.911) | | 0/57 (0.0%) | |
| Table S5. Comparison of Baseline Characteristics between Completers and Non-completers at 3-month Follow-up**Variable** | | | | **Completers (N=71)** | | **Non-completers (N=43)** | | **P-value** |
| Age (years) | | | | 63.62 \± 9.50 | | 62.00 \± 10.22 | | 0.4022 |
| BMI (kg/m^2^) | | | | 21.19 \± 1.29 | | 21.01 \± 1.22 | | 0.4533 |
| Sex (Male, %) | | | | 39 (54.9%) | | 34 (79.1%) | | **0.0163** |
| Any Comorbidity (%) | | | | 7 (9.9%) | | 9 (20.9%) | | 0.1703 |
| WBC (\times 10^9^/L) | | | | 6.36 \± 2.25 | | 6.77 \± 2.28 | | 0.3440 |
| CRP (mg/L) | | | | 13.21 \± 13.33 | | 12.44 \± 11.50 | | 0.7455 |
| PCT (ng/mL) | | | | 0.08 \± 0.09 | | 0.07 \± 0.08 | | 0.6035 |
| Hemoglobin (g/L) | | | | 123.51 \± 14.13 | | 126.65 \± 12.65 | | 0.2216 |
| Albumin (g/L) | | | | 36.54 \± 2.71 | | 36.36 \± 2.57 | | 0.7282 |
| Cavity Volume (mL) | | | | 184.61 \± 62.58 | | 169.95 \± 59.13 | | 0.2126 |
| ADL score | | | | 90.00 \± 13.94 | | 88.49 \± 14.94 | | 0.5928 |

Note: Values are Mean ± SD or n (%). P-values were calculated using t-tests for continuous variables and Chi-square tests for categorical variables*.*
